# Enduring Effects of Paternal Deprivation in California Mice (*Peromyscus californicus*): Behavioral Dysfunction and Sex-Dependent Alterations in Hippocampal New Cell Survival

**DOI:** 10.3389/fnbeh.2018.00020

**Published:** 2018-02-13

**Authors:** Erica R. Glasper, Molly M. Hyer, Terrence J. Hunter

**Affiliations:** ^1^Department of Psychology, University of Maryland, College Park, College Park, MD, United States; ^2^Program in Neuroscience and Cognitive Science, University of Maryland, College Park, College Park, MD, United States

**Keywords:** early-life environment, paternal deprivation, sex differences, cell survival, hippocampus

## Abstract

Early-life experiences with caregivers can significantly affect offspring development in human and non-human animals. While much of our knowledge of parent-offspring relationships stem from mother-offspring interactions, increasing evidence suggests interactions with the father are equally as important and can prevent social, behavioral, and neurological impairments that may appear early in life and have enduring consequences in adulthood. In the present study, we utilized the monogamous and biparental California mouse (*Peromyscus californicus*). California mouse fathers provide extensive offspring care and are essential for offspring survival. Non-sibling virgin male and female mice were randomly assigned to one of two experimental groups following the birth of their first litter: (1) biparental care: mate pairs remained with their offspring until weaning; or (2) paternal deprivation (PD): paternal males were permanently removed from their home cage on postnatal day (PND) 1. We assessed neonatal mortality rates, body weight, survival of adult born cells in the dentate gyrus of the hippocampus, and anxiety-like and passive stress-coping behaviors in male and female young adult offspring. While all biparentally-reared mice survived to weaning, PD resulted in a ~35% reduction in survival of offspring. Despite this reduction in survival to weaning, biparentally-reared and PD mice did not differ in body weight at weaning or into young adulthood. A sex-dependent effect of PD was observed on new cell survival in the dentate gyrus of the hippocampus, such that PD reduced cell survival in female, but not male, mice. While PD did not alter classic measures of anxiety-like behavior during the elevated plus maze task, exploratory behavior was reduced in PD mice. This observation was irrespective of sex. Additionally, PD increased some passive stress-coping behaviors (i.e., percent time spent immobile) during the forced swim task—an effect that was also not sex-dependent. Together, these findings demonstrate that, in a species where paternal care is not only important for offspring survival, PD can also contribute to altered structural and functional neuroplasticity of the hippocampus. The mechanisms contributing to the observed sex-dependent alterations in new cell survival in the dentate gyrus should be further investigated.

## Introduction

Offspring development is dependent on early bond formation with a caregiver (Rilling and Young, 2014). Lack of bond formation can result in impairments in behavior and neurodevelopmental disorders which may appear early in life (Japel et al., 1999) and persist into adulthood (Parker, 1979; Noorikhajavi et al., 2007; Tyrka et al., 2008). While the vast majority of our knowledge of parent-offspring relationships stem from mother-infant interactions (reviewed in Curley and Champagne, 2016), a few early human studies focused on the negative effects of paternal deprivation (PD) on offspring development (Green and Beall, 1962; Jensen et al., 1989). Increasing evidence from non-human animal studies suggests numerous adverse outcomes associated with PD, including dysregulated stress responses, impaired synaptic development in the prefrontal cortex, altered anxiety-like, social, and drug-seeking behaviors (Helmeke et al., 2009; Pinkernelle et al., 2009; Jia et al., 2011; Gos et al., 2014; Wang et al., 2017). Despite these advances in our knowledge, the underlying mechanisms of PD-related behavioral and neurobiological deficits remain unclear.

While fathers play a significant role in offspring care in many human societies (Kleiman and Malcolm, 1981; Hrdy, 2005), paternal, or biparental care, is rare in mammals and is observed in less than 6% of species examined (Kleiman and Malcolm, 1981). California mice (*Peromyscus californicus*) are a biparental species that are exclusively monogamous in the wild (Ribble, 1991), exhibit strong attraction and preference for the bonded mate over others (Gubernick and Nordby, 1993), and demonstrate significant paternal investment. Paternal California mice engage in many behaviors performed by the maternal female, including licking and grooming (LG), huddling, nest building, and pup retrieval (Dudley, 1974b; Gubernick and Alberts, 1987; Gubernick and Nelson, 1989; Gubernick and Nordby, 1993). This substantial investment in offspring care reduces offspring mortality and/or aids in development and growth (Dudley, 1974a; Gubernick and Alberts, 1987; Gubernick et al., 1993; Gubernick and Teferi, 2000). In the absence of the father, maternal females do not compensate for the missing paternal male (Dudley, 1974b)—an effect observed in other monogamous species as well (common degu, Helmeke et al., 2009; mandarin vole, Jia et al., 2009). Furthermore, as females of this species are highly aggressive towards conspecifics (reviewed in Steinman and Trainor, 2017), offspring care provided by multiple females is highly unlikely. Therefore, the California mouse is an excellent mouse model to investigate the consequences of PD on neurobiological outcomes.

Experiments using monogamous and biparental species suggest PD has long-lasting effects on hippocampal neurochemical systems (Wu et al., 2014; Tabbaa et al., 2017), as well as the structure and function of the hippocampus (Seidel et al., 2011; Braun et al., 2013). The hippocampus plays a key role in modulation of emotions (reviewed in Lucassen et al., 2014) and regulation of the stress response system (reviewed in Herman et al., 2016). The dentate gyrus of the hippocampus is heavily implicated in the mediation of anxiety-like behavior (Kheirbek et al., 2013; reviewed in Wu et al., 2015). More recently, a functional association between adult hippocampal neurogenesis and anxiety- and depressive-like behaviors has been demonstrated. A reduction in neurons (i.e., doublecortin positive cells; DCX+) is associated with stress-related anxiety and depressive behavior; a return to baseline DCX+ cell number results in normalization of anxiety- and depressive-like behaviors (Yun et al., 2016).

Evidence from studies using human subjects suggests that sexual dimorphisms in anxiety exist, with women largely more vulnerable than men (Kessler et al., 1994; McHenry et al., 2014). One likely underlying mechanism contributing to this sexual dimorphism in functionality of the hippocampus may be the regulation of adult neurogenesis (reviewed in Marques et al., 2016). Sex-dependent abnormalities in social- and reward-related behaviors have been observed in California mice following PD (Bambico et al., 2015) and PD increases anxiety-like behavior in adult mandarin voles (*Microtus mandarinus, Jia et al., 2009*). To what extent anxiety-like behavior and other hippocampus-related behaviors are regulated in a sex-dependent manner by PD in California mice is unknown. Therefore, the purpose of this study was to examine the interactions between sex and PD on the survival of adult born cells in the hippocampus and hippocampus-mediated behaviors, such as anxiety and passive-stress coping behavior, in young adult California mice.

## Materials and Methods

### Animals

Virgin male and female California mice (60–90 days of age) were obtained from the Peromyscus Genetic Stock Center (University of South Carolina, Columbia, SC, USA) or were descendants of mice bred in our colony. Mice were provided *ad libitum* access to food and water and were housed on a 16:8 reversed light/dark cycle (lights off at 11:00 h). Non-sibling males and females were paired, allowed to mate, and give birth to their first litter. Twenty-six mating pairs resulted in 43 total offspring (Table 1). An average of 1.64 ± 0.58 offspring per litter were produced. On postnatal day (PND) 1 (12:00 h), two experimental groups of offspring were formed by either leaving paternal males with their mate and offspring (biparental care) or removing paternal males from the home cage (PD). This resulted in the following groups of experimental offspring: biparental care (*n* = male: 11; female: 10) and PD (*n* = male: 14; female: 8). All surviving offspring were weaned on PND 35 and housed in same-sex groups of three (i.e., some same-sex non-siblings were housed together so that individual housing of mice could be avoided). This study was carried out in accordance with guidelines provided by the National Institutes of Health for the care and use of animals. The protocol was approved by the University of Maryland Institutional Animal Care and Use Committee.

**Table 1:** Litter size and number of primiparous California mouse mate pairs.

Litter size	Number of mated pairs
1	11
2	13
3	2

### Experimental Design

On PND 60, all biparentally-reared and PD offspring were administered an intraperitoneal injection of the DNA synthesis marker bromodeoxyuridine (BrdU; 200 mg/kg; Sigma-Aldrich, St. Louis, MO, USA; cat. no. B5002) to determine the extent to which sex and PD alter short-term survival of adult-born cells in the dentate gyrus of the hippocampus. On PND 65, all biparentally-reared and PD mice were tested for anxiety-like behavior on the elevated plus maze task (see below). On PND 67, all biparentally-reared and PD mice were assessed on a single trial version of the forced swim task, a behavioral task used to assess passive stress-coping behavior (see below). On PND 68, all mice were perfused and brain tissue was harvested in preparation for immunohistochemical processing (see below).

### Elevated Plus Maze Task

Mice were individually removed from their home cages, ~2 h after lights out, and placed in a holding cage for transportation to an adjacent behavioral room. After a 10-min acclimation period, mice were tested on the elevated plus maze under red-light illumination. The maze stood 75 cm above the floor with arms measuring 11.5 × 55 × 45 cm. Mice were placed in the center of the maze, facing an open arm, and observed for 5 min. Behavior was digitally recorded and analyzed with EthoVision^®^XT 11 behavioral tracking software (Noldus, Leesburg, VA, USA). Recordings were taken from a top-down view at a rate of 30 frames per second. Latency to enter the arms, duration of time spent in the arms, and number of arm entries were assessed as previously described (Glasper et al., 2015; Hyer et al., 2016). Duration of time spent in the open arms was calculated as total time spent in the open arms divided by the total time spent in both the open and closed arms, excluding the center, multiplied by 100 and presented as a percentage. Mice were returned to their holding cages immediately following the conclusion of testing and returned to the colony. Mice that froze for >40% of the time (Chauke et al., 2012) were excluded from the study (*n* = 7, across all groups).

### Forced Swim Task

The forced swim task was performed as previously described (Hyer and Glasper, 2017). Mice were transported to the red-light illuminated behavioral room, ~2 h after lights out, and acclimated as described above. This task consisted of placing mice in a Plexiglas cylinder (30 cm diameter, 43 cm deep) filled $\frac{3}{4}$ of the way with 23–25°C tap water for 5 min. Behavior was digitally recorded from a side view of the cylinder at 30 frames per second in an effort to distinguish between swimming and immobility behaviors (Bogdanova et al., 2013). Behavior during the task was analyzed with EthoVision^®^XT 11 behavioral tracking software (Noldus). The following behaviors were used to assess passive stress-coping behavior: % time immobile, latency to the first bout of immobility, and frequency of immobility bouts. Immobility was defined as mice remaining parallel to the surface of the water, only moving slightly to remain afloat. Swimming was defined as mice continuously moving paws and head. Following testing, mice were dried, warmed on a heating pad placed under their transportation cage, and returned to their home cage. Flipping behavior during the forced swim task greatly increases the likelihood that California mice will ingest water (unpublished observations); therefore, any mice that exhibited flipping behavior during the task were quickly removed and were excluded from all endpoints (*n* = 2, across all groups).

### Histological Procedures

On PND 68, ~2 h after lights out, mice were anesthetized using a ketamine–xylazine cocktail and transcardially perfused with 4% paraformaldehyde (PFA) in 0.1 M phosphate buffer, pH 7.0. Brains were dissected from the skull and postfixed in 4% PFA for at least 48 h at 4°C. Coronal sections (40 μm) were sliced throughout the rostrocaudal extent of the dentate gyrus on a vibrating microtome (Leica Microsystems, Chicago, IL, USA) into a bath of chilled 0.1 M phosphate-buffered saline (PBS), pH 7.5. Sections containing the dentate gyrus were identified using the Peromyscus brain atlas^1^.

### Immunoperoxidase Staining for BrdU

For BrdU peroxidase staining, a 1:12 series of sections were mounted onto glass Super Frost Plus slides (Fisher Scientific, Pittsburgh, PA, USA), dried, and pretreated by heating in 0.1 M citric acid, pH 6.0. Tissue was rinsed with PBS, incubated in trypsin for 10 min, denatured in 2 M HCL : PBS for 30 min, rinsed with PBS, incubated overnight in purified mouse anti-BrdU (1:200; BD Biosciences, San Jose, CA, USA; cat. no. 347580), incubated in biotinylated horse anti-mouse (1:200; Vector, Burlingame, CA, USA; cat. no. BA-2000) for 60 min, rinsed in PBS, incubated with avidin–biotin complex (Vector), rinsed with PBS, and then reacted in 0.01% diaminobenzadine with 0.003% H_2_O_2_. All slides were counterstained with cresyl violet, dehydrated, cleared with Citrisolv (Fisher Scientific), and coverslipped under Permount (Fisher Scientific).

### Data Analysis

Quantitative analysis was conducted on coded slides. The numbers of BrdU-labeled cells on every twelfth unilateral section throughout the rostrocaudal extent of the dentate gyrus (i.e., granule cell layer, subgranular zone, and hilus) were counted at 100× magnification under oil immersion on a Zeiss Primo Star light microscope (Zeiss, Thornwood, NY, USA) using a modified version of the optical fractionator method (West et al., 1991; Ngwenya et al., 2005). The simplified formula for the estimated total number of labeled cells was: N Σ Q × (1/ssf), which is the total number of labeled cells counted (N Σ Q) multiplied by the reciprocal of the section sampling fraction (1/ssf or 1/12; Leuner et al., 2009). Brightfield photomicrographs were taken with an AxioImager camera attached to a Zeiss microscope with a stage controller using neuroimaging software (Neurolucida, Williston, VT, USA). Images were cropped and optimized by adjusting brightness and color balance in Adobe Photoshop Creative Cloud 2014.2.2.

### Statistics

Data were analyzed using GraphPad Prism version 7.03 for Windows (GraphPad Software, La Jolla, CA, USA)^2^, unless otherwise noted. Survival to weaning was assessed via Log-rank (Mantel-Cox) Chi square analysis. Short-term cell survival was assessed by multiple *t*-test analysis and statistical significance was determined using the Holm-Sidak method. Two-way analysis of variance (ANOVA) was performed to assess main effects of sex and rearing condition on all behavioral endpoints (i.e., elevated plus maze, forced swim test), while three-way ANOVA was performed to assess main effects of sex, age and rearing condition on body weight using IBM^®^ SPSS^®^ Statistics (Version 24). Sidak’s multiple comparisons tests were performed following ANOVAs, when appropriate, and the multiplicity-adjusted *p-value* was reported for each comparison. Mean differences were considered statistically significant when *p* ≤ 0.05. For neuronal and behavioral analyses, final N sizes are reported within figure captions.

## Results

### Paternal Deprivation Decreases Neonatal Survival in California Mice

**Figure 1:**
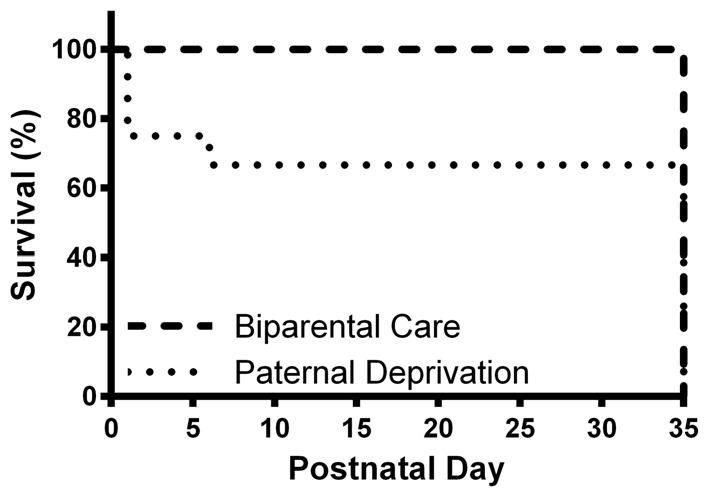
Paternal deprivation (PD) decreases survival to weaning in California mice. Offspring were born on postnatal day (PND) 0. On PND 1, fathers remained with mate and offspring (biparental care) or were permanently removed (PD). Offspring survival was assessed daily until weaning (PND 35). All California mouse offspring reared under biparental conditions survived to weaning (dashed lines). However, by PND 1, only ~75% of PD offspring (dotted lines) were observed alive. By PND 6, survival of PD offspring dropped to ~65% and remained constant until weaning.

We assessed the effects of PD on survival to weaning in *P. californicus* offspring. All (100%) biparentally-reared mice survived to weaning (PND 35; Figure 1). In contrast, PD mice displayed a marked and statistical decline in survival, with 66.67% surviving to PND 35 ($\chi_{\left(1,N\;=\;25\right)}^{2}$ = 4.96, *p* = 0.03). By the end of the dark cycle on PND 1, greater than 20% of PD offspring perished. Between PND 1 and PND 6, an additional 15% of PD offspring were found deceased. After PND 6, no additional PD deaths were observed.

### Paternal Deprivation Does Not Alter Growth of Offspring

**Figure 2:**
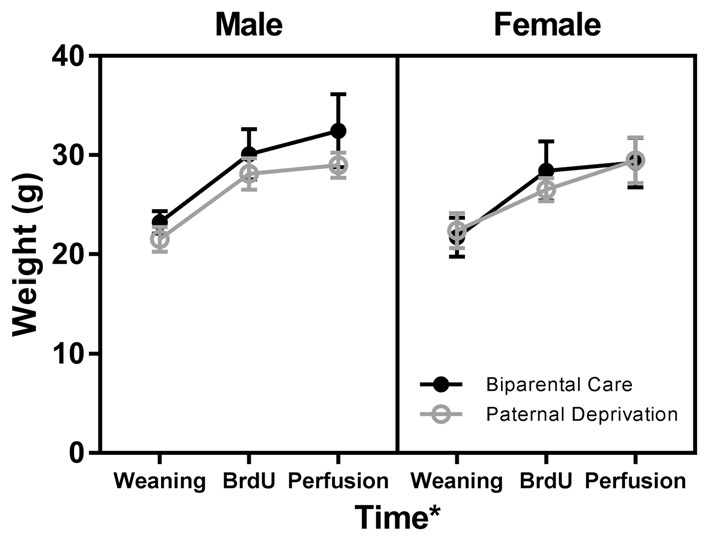
Paternal deprivation (PD), in California mice, does not alter body weight. Male and female California mice were reared by both parents (biparental care) or by the mother alone (PD) from postnatal day (PND) 1 until weaning. Body weight was assessed at weaning (PND 35), prior to BrdU injection (PND 60), and prior to perfusion (PND 68). Compared to weaning weight, all mice, regardless of rearing condition or sex, weighed more at the time of BrdU injection and at perfusion. Symbols represent mean ± SEM. *, main effect of time, *p* ≤ 0.05.

We investigated the effects of sex, rearing, and time on body weight at numerous points during the experiment: at weaning (PND 35), at the time of BrdU injection (PND 60), and immediately before perfusion (PND 68; Figure 2). No interaction between sex, rearing, and time on body weight was observed (*F*_(2,66)_ = 0.19, *p* = 0.83). No interactions between rearing and time (*F*_(2,66)_ = 0.10, *p* = 0.90), sex and time (*F*_(2,66)_ = 0.13, *p* = 0.88), or sex and rearing (*F*_(2,66)_ = 0.77, *p* = 0.38) on body weight were observed. No main effects of sex (*F*_(1,66)_ = 0.90, *p* = 0.35) or rearing (*F*_(1,66)_ = 1.69, *p* = 0.20) were observed. However, a main effect of time on body weight was observed (*F*_(2,66)_ = 16.40, *p* = 0.00). Compared to weaning weight, mice weighed more at time of BrdU injection (*p* = 0.00) and time of perfusion (*p* = 0.00). No difference in weight was observed between time of BrdU injection and time of perfusion (*p* = 0.53).

### Survival of Hippocampal Newborn Cells Is Reduced in Paternally-Deprived Female, but Not Male, Young Adult Offspring

**Figure 3:**
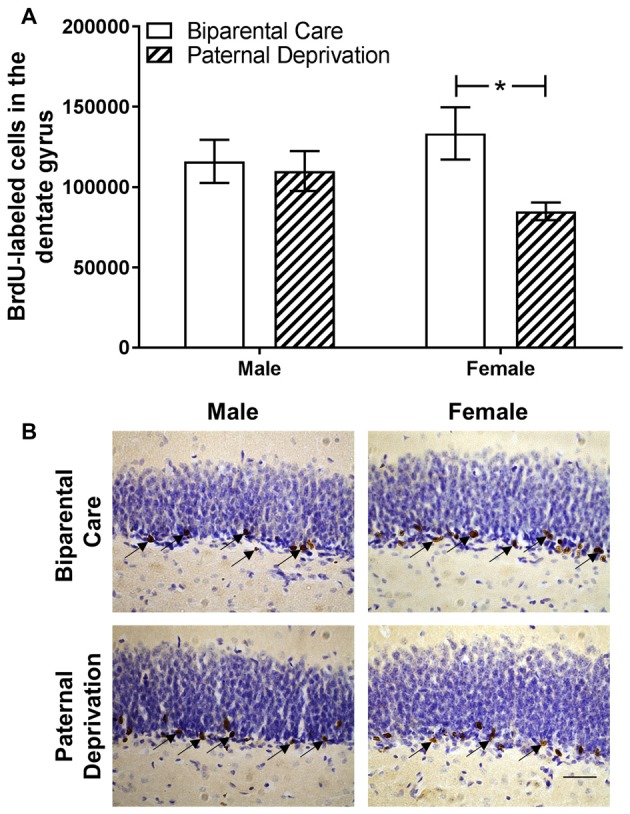
Paternal deprivation (PD) reduces new cell survival in the hippocampus of female, but not male, young adult California mouse offspring. **(A)** Short-term survival of bromodeoxyuridine (BrdU)-labeled cells was determined in California mouse young adult male and female offspring reared by both parents (biparental care) or reared by mother alone (PD) from postnatal day (PND) 1 to weaning PD. PD reduced the number of BrdU-labeled cells in the dentate gyrus of female, but not male, offspring. N sizes: biparental male, 6; biparental female, 6; PD male, 9; PD female, 6. Bars represent mean ± SEM. *p ≤ 0.05. **(B)** Representative photomicrographs (40× oil magnification) of BrdU-labeled cells in the dentate gyrus of the hippocampus. Scale bar = 40 μm. Arrows point to BrdU-labeled cells.

The effects of PD on survival of adult born cells in the dentate gyrus of the hippocampus were investigated in young adult male and female offspring (Figure 3). Among males, rearing condition did not alter number of BrdU-labeled cells in the dentate gyrus (*t*_(23)_ = 0.34, *p* = 0.74). Among females, PD resulted in fewer BrdU-labeled cells in the dentate gyrus compared to biparental care (*t*_(23)_ = 2.53, *p* = 0.02).

### Sex and Paternal Deprivation Alter Elevated Plus Maze Behavior in Young Adult Offspring

**Figure 4:**
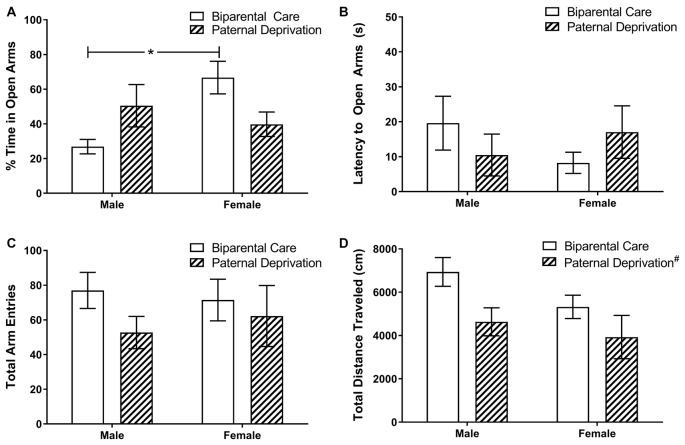
Classic indices of anxiety-like behavior in the elevated plus maze are not altered by paternal deprivation (PD) among young adult California mice. Male and female California mice were reared by both parents (biparental care) or by the mother alone (PD) from postnatal day (PND) 1 until weaning. On PND 65, mice were tested on the elevated plus maze task for 5 min. **(A)** Among biparentally-reared offspring, females spent considerably more time on the open arms than males. PD did not alter time spent on the open arms. *p ≤ 0.05. **(B,C)** Neither sex, nor rearing, altered latency to enter the open arms or total arm entries. **(D)** Irrespective of sex, total distance traveled within the elevated plus maze was reduced by PD. N sizes: biparental care male; 4; biparental care female, 7; PD male, 7; PD female, 5. Bars represent mean ± SEM. #, main effect of PD.

We examined the effects of sex and PD on anxiety-like behavior in young adult offspring, as measured by performance on the elevated plus maze task. In % time spent on the open arms (Figure 4A), a significant interaction between rearing and sex was observed (*F*_(1,19)_ = 5.89, *p* = 0.03). Among biparentally-reared mice, females spent considerably more time on the open arms than males (*p* = 0.03); however, this sex effect was not observed in PD mice (*p* = 0.71). No main effect of sex (*F*_(1,19)_ = 1.95, *p* = 0.18) or rearing (*F*_(1,19)_ = 0.03, *p* = 0.87) was observed in % time on the open arms. Latency to enter the open arms (Figure 4B) was not altered by rearing (*F*_(1,19)_ = 0.000, *p* = 0.98) or sex (*F*_(1,19)_ = 0.16, *p* = 0.69) and no interaction between rearing and sex was observed (*F*_(1,19)_ = 2.22, *p* = 0.69). Total arm entries (Figure 4C) were not altered by rearing (*F*_(1,19)_ = 1.69, *p* = 0.21) or sex (*F*_(1,19)_ = 0.02, *p* = 0.88) and no interaction between rearing and sex was detected (*F*_(1,19)_ = 0.34, *p* = 0.57). In total distance traveled (Figure 4D), a main effect of rearing (*F*_(1,19)_ = 6.40, *p* = 0.02), but not sex (*F*_(1,19)_ = 2.53, *p* = 0.13), was observed. Overall, PD decreased the total distance traveled during the elevated plus maze task. No interaction between rearing and sex (*F*_(1,19)_ = 0.39, *p* = 0.54) was observed.

### Paternal Deprivation and Sex Alter Passive Stress-Coping Behavior in Young Adult Offspring

**Figure 5:**
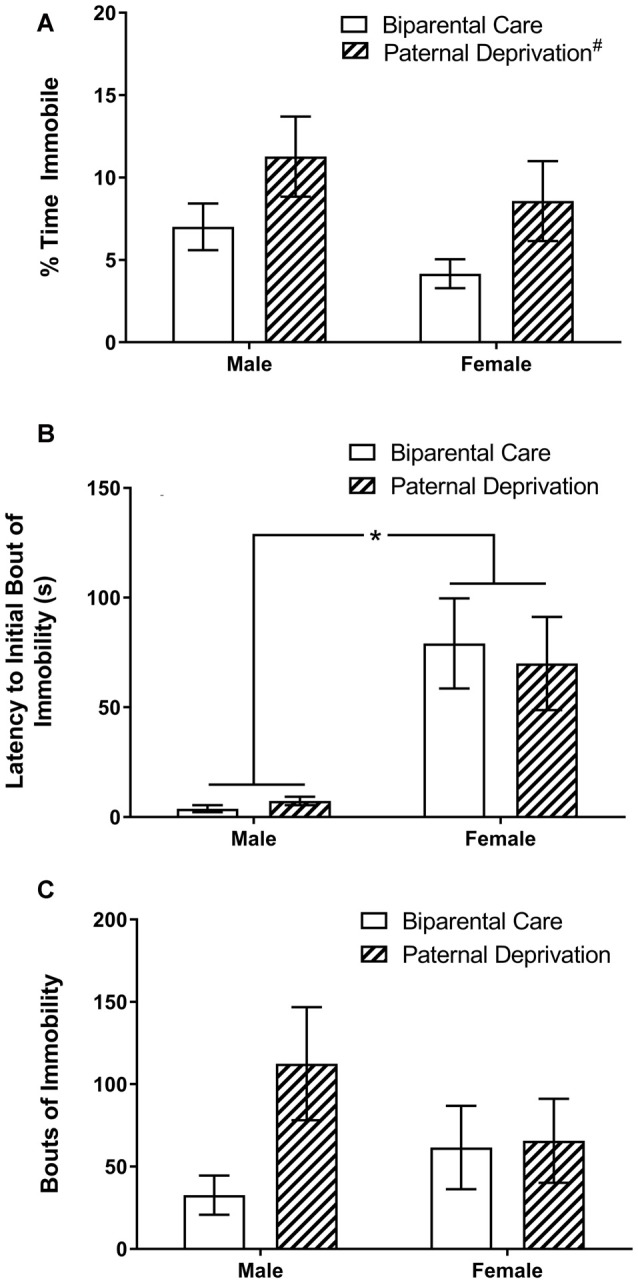
Both rearing and sex alter indices of passive stress-coping behavior during the forced swim test in young adult California mice. Male and female California mice were reared by both parents (biparental care) or by the mother alone [paternal deprivation (PD)] from postnatal day (PND) 1 until weaning. On PND 67, mice underwent a 5 min forced swim test. **(A)** Overall, PD increases % time immobile. #, main effect of PD. **(B)** Irrespective of rearing, males display immobility faster than females. *p ≤ 0.05. **(C)** Neither sex, nor rearing, alter bouts of immobility. N sizes: biparental care male, 8; biparental care female, 7; PD male, 5; PD female, 6. Bars represent mean ± SEM.

We determined the effects of PD and sex on passive stress-coping behavior, during the forced swim task, in young adult male and female offspring. In % time immobile (Figure 5A), a main effect of rearing (*F*_(1,22)_ = 4.72, *p* = 0.04), but not sex (*F*_(1,22)_ = 1.93, *p* = 0.18), was observed. Overall, PD increased % time spent immobile during the forced swim task. No interaction between rearing and sex was observed in % time immobile (*F*_(1,22)_ = 0.00, *p* = 0.97). Latency to the first bout of immobility (Figure 5B) was significantly altered by sex (*F*_(1,22)_ = 29.54, *p* < 0.00) but not rearing (*F*_(1,22)_ = 0.05, *p* = 0.83). Males, irrespective of rearing, displayed passive stress-coping behavior (i.e., floating) faster than females. No interaction between rearing and sex was observed (*F*_(1,22)_ = 0.25, *p* = 0.62). Bouts of immobility (Figure 5C) were also not altered by rearing (*F*_(1,22)_ = 2.72, *p* = 0.11) or sex (*F*_(1,22)_ = 0.12, *p* = 0.73), and no interaction between rearing and sex was observed (*F*_(1,22)_ = 2.22, *p* = 0.15).

## Discussion

In the present study, we demonstrated that PD in *P. californicus*, a biparental mouse species, is associated with reduced survival during early postnatal development, sex-dependent deficits in hippocampal structural plasticity, reduced exploratory behavior, and impaired stress coping in young adulthood. PD results in ~35% decrease in offspring survival to weaning. Of those offspring that survive to weaning, no differences in body weight are detected; however, a sex-dependent decrease in the number of adult-born cells is observed in the dentate gyrus of the hippocampus. Specifically, PD females, but not males, exhibit reduced short-term survival of newborn cells in the dentate gyrus. Additionally, PD decreases exploratory behavior, but not classic anxiety-like behaviors, during the elevated plus maze task. Notably, and for the first time shown here, PD increases some measures of passive stress-coping behavior during the forced swim task (i.e., % time immobile). Together, these findings suggest that the lack of paternal care, in a biparental species, may contribute to long-lasting effects on structural plasticity and behavioral function of the hippocampus.

California mouse fathers spend more time interacting with offspring during the early, compared to late, postnatal period (Bester-Meredith et al., 1999). Here, removing the paternal male from the home cage resulted in a significant decline in early (i.e., PND 1) postnatal survival. As previously mentioned, California mice are an excellent model of biparental care given the significant paternal care provided by California mouse fathers. Male and female California mice parents spend similar amounts of time in the nest (Dudley, 1974a) and aside from nursing, parental behaviors performed on PND 1 are shared equally by both the mother and father. Specifically, California mouse fathers and mothers spend equal amounts of time in the nest as well as equivalent durations of time in physical contact with the pups (Gubernick and Alberts, 1987). On the whole, the early parental behaviors performed by fathers are similar to that performed by mothers, however, fathers do perform more non-anogenital licking of pups than mothers on PND 1 (Gubernick and Alberts, 1987).

When dead pups were observed, they were either cannibalized or found unaltered outside of the nest, an outcome previously reported in studies of California mice (Gubernick et al., 1993). Pup death may be mediated, in part, by the dam’s response to the absence of her mate. It is not uncommon for maternal California mice to cannibalize or withhold care from young following mate disappearance (Gubernick et al., 1993). A rapid termination of the dam’s reproductive investments following the removal of the mate may reflect the inability to successfully rear pups alone, an effect also observed in the monogamous, biparental Djungarian hamster (*Phodopus cambelli*); all pups observed deceased 3 days postpartum if paternal male is removed (Wynne-Edwards, 1987; Wynne-Edwards and Lisk, 1989). In the current study, PD offspring survived if they lived to PND 6. Interestingly, if California mice parents decide to forgo offspring care, pups are observed deceased 2–5 days following birth (Cantoni and Brown, 1997). It should also be noted that offspring survival is decreased even when males are removed several days before the birth of pups (Gubernick et al., 1993), therefore the increase in offspring mortality is likely not a result of experimenter handling or nest disruption.

Increased pup death may also be due to problems related to thermoregulation and/or metabolism. Thermoregulation, in California mice pups, is related to the presence of the father (Dudley, 1974b); individual California mice offspring are ectothermic prior to PND 15 (Gubernick, 1987). Given that most of the male’s early parental care is in the form of huddling over pups (Gubernick and Alberts, 1987), direct male care may enhance offspring survival by providing warmth, as previously described (Dudley, 1974a). Heat transfer may be even more necessary under harsh environmental conditions, such as cold temperatures and/or when foraging for food is necessary (Gubernick et al., 1993; Wright and Brown, 2002; Bredy et al., 2007). It is likely that under harsh laboratory conditions, our survival rate would be lower than what is reported in the current study. In addition to thermoregulation, problems associated with metabolism may contribute to offspring mortality. Following mate removal, California mouse mothers stop lactating 5–28 days later (Gubernick and Teferi, 2000). It is possible that PD offspring receive less nourishment than biparentally-reared offspring, which may ultimately contribute to increased mortality. Future studies are necessary to determine to what extent increased California mouse mortality, following the removal of the paternal male, is a result of the direct absence of paternal care, since California mice dams do not overcompensate for their partners’ absence (Dudley, 1974b), or indirect effects of altered maternal care. Recent evidence from our lab has demonstrated no differences in pup retrieval between multiparous California mouse mothers rearing pups with or without her mate (Madison et al., 2017). Pup retrieval is only one of many maternal behaviors, therefore a thorough analysis of parental behavior should be performed.

A sex-dependent effect of PD on the short-term survival of adult born cells in the dentate gyrus of the hippocampus was observed. Specifically, PD females exhibited a marked decline in the number of BrdU-labeled cells, compared to females reared by both parents. No effect of PD was observed in the short-term survival of adult born cells in males. While the phenotype of these 8-day old cells was not assessed in the current study, doublecortin (DCX) is expressed in the majority (~89%) of 1-week old BrdU-labeled cells in the hippocampus of young adult mice (Snyder et al., 2009). DCX is expressed in young neurons as well as neuronal precursors and plays key roles in neuronal maturation (Brown et al., 2003; Kerjan et al., 2009). This sex-dependent effect of PD on short-term cell survival in the dentate gyrus of the hippocampus aligns with sex-dependent effects of PD on neuroendocrine regulation and brain neurochemistry observed in biparental rodents, including the California mouse. Serum corticosterone and adrenocorticotrophin concentrations are increased in adult female, but not male, mandarin voles exposed to PD (Wu et al., 2014). Since stress and elevated glucocorticoids have been repeatedly shown to inhibit new cell production and survival (reviewed in Mirescu and Gould, 2006), the decreased cell survival in our PD females, but not males, may reflect baseline differences in serum corticosterone. This was not assessed in our current study and should be further explored. Decreased hippocampal glucocorticoid receptor (GR) and brain-derived neurotrophic factor (BDNF) have been shown in female, but not male, mandarin voles (Wu et al., 2014). Additionally, an attenuation in basal activity of low-spiking medial prefrontal cortex pyramidal cells has been observed in female, but not male, California mice (Bambico et al., 2015). In male, but not female, degus (*Octodon degus*), PD results in early (i.e., time of weaning) deficits in dendritic plasticity of the orbitofrontal cortex—an effect that is no longer apparent in adulthood (Helmeke et al., 2009). It is unknown whether similar developmental trajectories exist in hippocampal structural plasticity in male California mice.

It is important to note that other models of early-life stress, (i.e., disrupted maternal care) in uniparental species, like mice and rats, results in sex-dependent alterations to hippocampal neuroplasticity. Twenty-four hours of maternal deprivation on PND 3 does not alter anxiety-like behavior, cognitive function, or adult neurogenesis in 12–17 week old female rats (Loi et al., 2017) but does lead to accelerated maturation of synaptic plasticity in male rats (Derks et al., 2016). At weaning (PND 21), maternal deprivation results in increased immature neuron survival (i.e., DCX+ cells) in male rats and decreased DCX+ cells in female rats. This effect was likely not driven by enduring sex-dependent changes in maternal behavior following maternal deprivation on PND 3 (Oomen et al., 2009). Maternal deprivation ~1 week later (i.e., PND 9) immunologically primes hippocampal synapses of male, but not female, juvenile rats (Viviani et al., 2014); reduced anxiety-like behavior and increased risk taking behaviors are observed in females only (Mela et al., 2015). Females may be more resilient than males to the effects of early-life stress (Walker et al., 2011), as early life nest and bedding disruption models describe enduring negative consequences in male mice only, including increased basal corticosterone concentrations, decreased spatial and object recognition memory, and decreased hippocampal adult neurogenesis (Rice et al., 2008; Naninck et al., 2015). To what extent similar sex-dependent observations occur, as a result of maternal deprivation, in a biparental mammalian species has yet to be investigated. Interestingly, in the biparental zebra finch (*Taeniopygia guttata*), maternal deprivation results in hyperresponsivity to stress and altered mRNA levels of GR and mineralocorticoid receptors in the hippocampus, cerebellum, and hypothalamus (Banerjee et al., 2012).

To what extent the lack of direct paternal care mediates the observed sex-dependent effects on new cell survival is unclear, however, it is conceivable that differential distribution of parental care may play a factor. California mouse fathers engage in more pup licking than mothers; however, fathers spend more time licking non-anogenital regions compared to mothers (Gubernick and Alberts, 1987). In rats, mothers engage in more anogenital licking of male, compared to female, offspring (Richmond and Sachs, 1984). This attentional bias toward male offspring could have long-term consequences on development (Moore and Power, 1992). While this bias in anogenital licking has not been reported in California mice, it is possible that male offspring receive more direct care from the mother in the form of anogenital licking, thereby providing more parental care, thus preventing a decline in hippocampal structural plasticity. More detailed analysis of home cage parental behavior following PD may shed light on this possibility given that maternal deprivation on PND 3 results in greater LG on PND 4 in rat offspring, with males receiving more attention than females (Oomen et al., 2009); however, both the sex difference in LG and overall increase in LG disappears by PND 5. To what extent similar findings are observed following PD in California mice should be further explored.

We did not observe an overall anxious phenotype among PD offspring. During elevated plus maze testing, classical indices of anxiety like behavior (i.e., reduced % time in the open arms, increased latency to enter open arms; Komada et al., 2008) were not observed following PD. However, exploratory behavior (i.e., total distance traveled) was reduced in both male and female PD offspring, compared to offspring receiving biparental care. A reduction in locomotor activity, without altered anxiety-like behavior, has been observed during assessments of anxiety-like behavior in PD mandarin voles (Jia et al., 2009; Tabbaa et al., 2017). In fact, reduced locomotor activity following PD has been observed in both rodent and non-human primate species (Dettling et al., 2002; Cao et al., 2014). Collectively, these studies of PD in various mammalian species suggest that decreased exploratory behavior may be indicative of an anxious phenotype (Kõks et al., 1997) that may complicate more traditional indices of anxiety-like behavior on the elevated plus maze. Despite the lack of a sex-dependent effect in anxiety-like behavior within the PD group, contrasted with the effect observed within the biparental care group, restraint should be taken when interpreting the effects of PD on anxiety-like behavior when exploration is a primary component of the behavioral task (e.g., elevated plus maze).

PD, independent of sex, resulted in increased total time spent immobile during the forced swim task. This is the first demonstration, to our knowledge, of increased passive stress-coping behavior following PD. Chronic physical and psychological stressors significantly alter regulation of neuroendocrine systems and reorganize brain regions, like the hippocampus, which are highly responsive to stress hormones (i.e., corticosterone; reviewed in de Kloet and Molendijk, 2016). It is plausible that PD altered neuroendocrine regulation, yet this was not assessed in the current study. Increased basal corticosterone has been observed throughout the postpartum period (Wang et al., 2014) and at weaning (Wang et al., 2012) in mandarin vole offspring following removal of the paternal male on PND 0 (i.e., day of birth). Mice with a history of stress, followed by exposure to the forced swim task, exhibit upregulation of genes in the hippocampus that are involved in chromatin modification and epigenetics (e.g., BDNF and GR). The altered expression of some of these genes can be long-lasting (Gray et al., 2014; Hashikawa et al., 2015) and may underlie immobility behavior during the forced swim task (De Pablo et al., 1989; Campus et al., 2015). Latency to immobility, or floating, is considered a main outcome measure of the forced swim task. In the current experiment, the time from placement in the cylinder to the first bout of immobility was markedly faster among males than females. This effect was independent of rearing. However, the total number of immobility bouts did not differ as a result of sex. Therefore, although male California mice exhibited earlier passive stress-coping behavior than females, this sex difference did not influence global performance in the forced swim task.

In summary, our findings highlight the consequences of PD in a biparental rodent species, the California mouse. Removal of the father was associated with reduced structural plasticity among female mice and generalized deficits in exploratory and passive-stress coping behaviors. In humans, quality, rather than continuity, of parental care is associated with impaired behavioral dysfunction (i.e., depression; Parker, 1979). Given that maternal California mice do not compensate for missing paternal contributions (Dudley, 1974a; Bester-Meredith and Marler, 2003), the quality of care received by PD offspring may be reduced, resulting in enduring effects on hippocampal neuroplasticity and even survival. Mechanisms underlying sex-differences in short-term survival should be explored. Additionally, future studies should investigate to what extent these findings are a direct result of paternal removal or an indirect result of altered maternal care following mate removal.

## Author Contributions

ERG: substantial contribution to the conception of the work; critically revising the work for intellectual content. ERG and MMH: substantial contribution to the design of the work. MMH and TJH: acquisition of data for the work. ERG, MMH and TJH: analysis of the data for the work, interpretation of the data for the work and final approval of the version to be published. ERG and TJH: drafting the work. ERG: critically revising the work for intellectual content.

## Conflict of Interest Statement

The authors declare that the research was conducted in the absence of any commercial or financial relationships that could be construed as a potential conflict of interest. The reviewer FRD and handling Editor declared their shared affiliation.

## Footnotes

^1^BrainMaps: An interactive multiresolution brain atlas. Available online at: http://brainmaps.org.

^2^www.graphpad.com
